# Small Molecule Inhibitors as Countermeasures for Botulinum Neurotoxin Intoxication

**DOI:** 10.3390/molecules16010202

**Published:** 2010-12-30

**Authors:** Bing Li, Norton P. Peet, Michelle M. Butler, James C. Burnett, Donald T. Moir, Terry L. Bowlin

**Affiliations:** 1 Microbiotix, Inc., One Innovation Drive, Worcester, MA 01605, USA; 2 Target, Structure-Based Drug Discovery Group, SAIC-Frederick, Inc., National Cancer Institute at Frederick, 1050 Boyles Street, Frederick, MD 21702, USA; E-Mail: burnettjames@mail.nih.gov (J.C.B.)

**Keywords:** botulinum neurotoxin, inhibitor, drug discovery

## Abstract

Botulinum neurotoxins (BoNTs) are the most potent of known toxins and are listed as category A biothreat agents by the U.S. CDC. The BoNT-mediated proteolysis of SNARE proteins inhibits the exocytosis of acetylcholine into neuromuscular junctions, leading to life-threatening flaccid paralysis. Currently, the only therapy for BoNT intoxication (which results in the disease state botulism) includes experimental preventative antibodies and long-term supportive care. Therefore, there is an urgent need to identify and develop inhibitors that will serve as both prophylactic agents and post-exposure ‘rescue’ therapeutics. This review focuses on recent progress to discover and develop small molecule inhibitors as therapeutic countermeasures for BoNT intoxication.

## 1. Introduction

Botulinum neurotoxins (BoNTs), secreted by the anaerobic spore-forming bacterial *Clostridia* species *botulinum, baratii, and butyricum,* are the most poisonous of known biological toxins [1,2], and as a result are listed as category A biothreat agents by the United States Centers for Disease Control and Prevention (CDC). BoNTs can be easily produced and may be delivered by either aerosol route, [2,3,4] or through contamination of the food supply. Consequently, these toxins represent a serious threat to both military personnel and civilians [5,6,7]. Moreover, since both BoNT/A (Botox™) and BoNT/B (Myobloc™) are available commercially, and are now used for cholinergic nerve and muscle dysfunction therapy, as well as cosmetic treatments [8,9,10,11,12,13,14,15,16], it is likely that overdose, misuse and/or adverse side effects [17] may result in systemic toxin exposure. The currently available BoNT toxoid vaccine, as well as experimental preventative antibodies, cannot counter these toxins in the neuronal cytosol. This is an important point, as it is likely that individuals will seek medical attention only after clinical symptoms of intoxication manifest (*i.e.*, life-threatening paralysis). Currently, critical care mechanical ventilation is the only treatment option once neurons have been intoxicated and diaphragm muscles cease to function. However, long-term mechanical ventilation would be impractical for the treatment of a large population of intoxicated individuals. Therefore, there is an urgent need to identify and develop low molecular weight, non-peptidic inhibitors that will serve as both prophylactic agents and post-exposure ‘rescue’ therapeutics. 

There are seven botulinum neurotoxin (BoNT) serotypes (A-G), which possess different tertiary structures and significant sequence divergence. Of the seven BoNT serotypes, A, B, and E are known to cause human botulism [18,19], with BoNT/A and BoNT/B exhibiting the longest durations of activity in the neuronal cytosol (*i.e.*, from several weeks to months, depending on the severity of the poisoning [20,21,22]). Hence, the vast majority of research to develop inhibitors to counter BoNT intoxication post-neuronal internalization has focused on the BoNT/A and BoNT/B light chains (LCs). Once inhaled into the lungs or ingested into the gastrointestinal tract, BoNTs are transcytosed across the respiratory epithelium or mucosa into the blood stream, where they can enter the intracellular space prior to accessing peripheral cholinergic nerve endings. Structurally, BoNTs are synthesized as single polypeptide chains that undergo bacterial or host-mediated cleavage resulting in a 100 kDa heavy chain (HC) component and a 50 kDa light chain (LC) component. These two components, which compose the biologically active holotoxin, are connected by a disulfide bridge until reaching the reducing cytosolic environment of the neuronal target cells [23,24]. The LC is a zinc-dependent endopeptidase. The intoxication of cells involves a stepwise sequence of cell surface binding, receptor-mediated endocytosis, pH-induced translocation, and cytosolic metalloendoprotease activity [24]. The HC serves as a delivery system for the proteolytic LC by binding to neurons and transporting the LC into the cytosol via endosomes. Each BoNT LC cleaves a component of the soluble N-ethylmaleimide-sensitive factor attachment protein receptor (SNARE) proteins [25,26], which are responsible for transporting acetylcholine into neuromuscular junctions. BoNT serotypes A and E cleave SNAP-25 (synaptosomal-associated protein (25 kDa)) [27], serotypes B, D, F, and G cleave VAMP-2 (vesicle-associated membrane protein, also referred to as synaptobrevin) [28,29,30,31,32], and serotype C cleaves both SNAP-25 and syntaxin 1 [33]. BoNT-mediated cleavage of any one of the three SNARE proteins terminates the function of autonomic nerves via the inhibition of acetylcholine release, which produces flaccid paralysis. Once diaphragm muscles are affected, suffocation results.

## 2. Crystal Structures of Botulinum Neurotoxins

**Figure 1:**
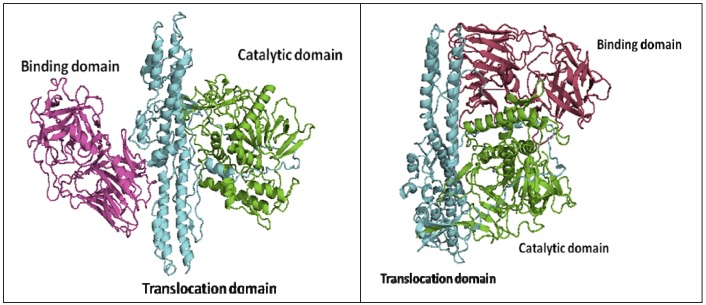
Cartoon representations of two types of three-domain organizations of BoNTholotoxins, (**a**) BoNT/A, left panel, and (**b**) BoNT/E, right panel. The two structures were obtained from the Protein Data Bank (PDB codes: 3BTA for BoNT/A and 3FFZ for BoNT/E).

The structures of BoNT proteins have been fairly well characterized. The crystal structures of the BoNT/A, B, and E holotoxins have been reported [23,34,35], and a handful of LC crystal structures and receptor binding domains of BoNT HCs are also available [36,37,38,39,40,41,42,43]. The BoNT proteins contain three functional domains: the binding domain, the translocation domain, and the catalytic domain. The LC folds into the catalytic domain and functions as a Zn-dependent endopeptidase that cleaves SNARE proteins in the neuronal cytosol. The C-terminus of the HC forms the binding domain, which targets the cell surface, while the N-terminus of the HC is involved in the translocation of the toxin across the neuronal membrane [44,45,46] (Figure 1). Based on sequence and functional similarity, it was originally believed that the three-dimensional structures of BoNTs would also be similar. Indeed, the structures of the individual functional domains in serotypes BoNT/A, B and E are similar; however, the overall domain arrangements are different [35]. In the BoNT/A and BoNT/B holotoxins, the three domains are arranged in a linear fashion, with the translocation domain in the center (Figure 1, left panel). However, in the BoNT/E holotoxin, both the binding domain and the catalytic domain are on the same side of the translocation domain, and all three domains mutually interact with one another (Figure 1, right panel). This unique association may result in the faster rate of internalization and translocation observed for the BoNT/E, and thus explains the faster intoxication rate of BoNT/E with respect to other BoNT serotypes.

Comparison of the x-ray structures of the BoNT/A holotoxin and the BoNT/B holotoxin indicate that the enzymes’ translocation domain protective belts, which wrap around the LCs of both holotoxins, possess different orientations. Specifically, in the BoNT/A, the protective belt completely obstructs the active site and inhibits substrate binding. Thus, the BoNT/A LC is catalytically active only after translocation into the neuronal cytosol and dissociation from the HC. Experimental evidence indicates that the BoNT/A LC, after separation from the rest of the holotoxin, is the actual active component [47]. In contrast, the orientation of the BoNT/B protective belt allows for active site access relative to the BoNT/A holotoxin [39], making the BoNT/B LC catalytically active prior to reduction of the disulfide bond.

**Figure 2:**
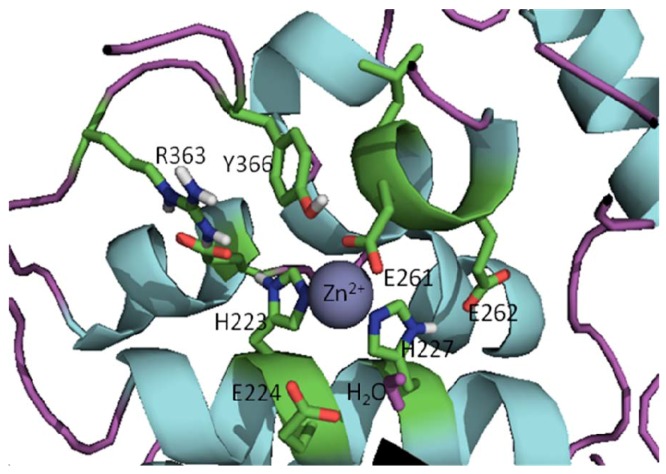
Active site of the BoNT/A LC (prepared from PDB code 2IMC) [55].

The active sites of BoNT LCs contain a common HEXXH (X is any amino acid) zinc-binding motif with two His residues and one Glu residue ligating the zinc [30,31,32], the catalytic water molecule provides the fourth zinc ligand (the structure of active site of the BoNT/A LC is shown in Figure 2). All seven BoNT serotype LCs contain one zinc atom, with the exception of the BoNT/C LC, which possesses two zinc atoms [48]. Superimposition of the x-ray structures of the catalytic clefts of the BoNT/A, B and E LCs indicates that their compositions and geometries are essentially identical [38]. Interestingly, BoNT LC sequence identities range from 31-59%, while sequence similarities range from 52-75% [49]. However, even though the active sites of BoNT LCs are highly homologous, serotype-specific inhibitors, which preferentially target different LC active sites, have been identified [50,51,52,53,54].

The seven BoNT LC serotypes exhibit unique substrate selectivity and cleavage site specificity. Hence, the virtually identical structures of the active sites of the neurotoxins suggest that substrate recognition is not dictated by the enzymes’ catalytic clefts [38]. Rather, and as evidenced by the crystal structure of the BoNT/A LC:SNAP-25 complex which comprises 4,840 Å^2^ of the enzyme-substrate interface area, the substrate recognition locations of BoNT LCs are discontinuous and distal to the catalytic site. Further evidence supporting this hypothesis is the large size of the substrate recognition requirement for the BoNT/A LC (*i.e.*, a minimum of 17 substrate amino acids are required for SNAP-25 cleavage) [56], which is unusual for metalloproteases. Based on the BoNT/A LC:SNAP-25 complex, two exosites, termed the a-exosite and the b-exosite, are required for substrate recognition [38]. In addition, a Cys165 site (we have termed it as the ¡-exosite), which is adjacent to the active site, has also been proposed for small molecule ligand binding [57]. The putative exosites of the BoNT/F LC, which were identified based on the crystal structure and molecular modeling, are different from that of the BoNT/A LC and the BoNT/B LC [41]. In general, the substrate selectivity and cleavage site specificities of the BoNT proteases have provided the bases for the discovery and development of serotype specific BoNT inhibitors.

## 3. Approaches to BoNT Inhibition

To counter the BoNT threat, several different approaches are currently being explored. While vaccines will likely play a role in biodefense [58,59], the development of therapeutic approaches that are effective both pre- and post-exposure are essential. In particular, vaccines are useless for the post-exposure protection of previously unvaccinated individuals, and the identification and inoculation of all members of large, at-risk populations prior to exposure is problematic. Therapeutic approaches under development include the following: (a) anti-BoNT antibodies, with the most effective strategy involving the simultaneous administration of three monoclonal antibodies. The antibodies bind the BoNT/A with non-overlapping reactivity, and provide potent protection against toxin challenge in mice [60]; (b) soluble versions of the BoNT/B and the BoNT/G receptors (“receptor decoys”),which function as anti-toxins in cell culture and in mice [61,62] (however, in the case of the BoNT/B, the anti-toxin effect requires the co-administration of gangliosides); and (c) small molecule approaches to BoNT inhibition. This final category is the focus of this review.

## 4. Broad-Spectrum Small Molecule BoNT Inhibitors

Numerous attempts to develop broad spectrum antagonists of BoNTs have met with limited success. One approach is to inhibit toxin-cell interactions by targeting carbohydrates in general, and sialic acid in particular, on cellular receptors. This approach is based on the premise that the cellular receptors for the toxins may not be identical, but may possess certain elements of commonality. Lectins, which are large glycoproteins that are highly specific for their sugar moieties, and which exert their effects by preventing the binding of the BoNT HC to the membrane receptor [63], were found to antagonize several serotypes. Finally, no small molecules that inhibit toxin-cell receptor interactions have been identified. 

**Figure 3:**
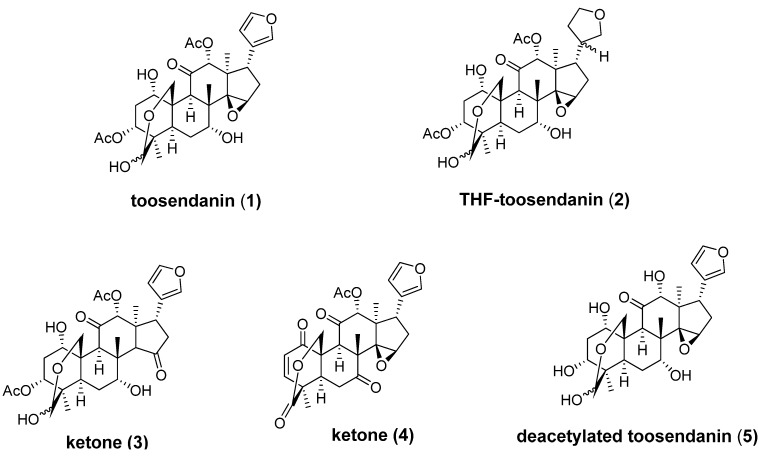
Chemical structures of toosendanin (**1**) and its analogs.

BoNT-induced muscle paralysis involves holotoxin translocation and subsequent release from an acidic endosome. In the early 1980s, the triterpenoid toosendanin (**1**, Figure 3), was reported to protect monkeys against BoNT/A, BoNT/B and BoNT/E-induced death in a dose-dependent fashion [64,65,66,67]. In a spinal cord cell-based assay, toosendanin completely inhibits SNAP-25 cleavage at concentrations above 200 nM, and partial inhibition can be observed with concentrations as low as 8 nM for BoNT/A and 40 nM for BoNT/E. Single molecule channel experiments have demonstrated that toosendanin exhibits an unprecedented dual mode of action within the protein-conducting channel, acting both as a cargo-dependent inhibitor of translocation and as a cargo-free channel activator [68]. To elucidate the mechanistic nature of its anti-BoNT properties, several toosendanin analogs have been prepared by semisynthetic approaches (Figure 3) [69,70]. Only the THF-toosendanin analog **2** exhibited similar activity, indicating that the furan ring of toosendanin can be modified. However, the epoxide moiety on the five-membered-ring still seems to be important for anti-BoNT activity, as replacement of the epoxide moiety with a thermodynamically more stable ketone resulted in inactive compound **3**. Ketone **4** and deacetylated compound **5** also lack activity against BoNTs. 

**Figure 4:**
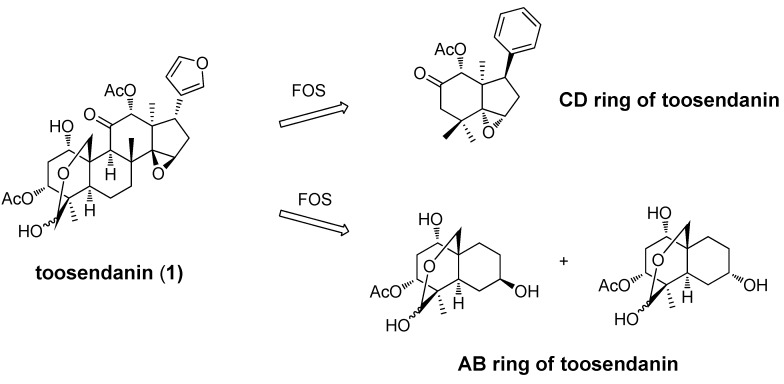
AB-ring and CD-ring fragments generated from a function-oriented synthesis (FOS) strategy.

The total synthesis of toosendanin (**1**) and its analogs would allow for a more thorough assessment of the structure-activity relationships (SARs) associated with this chemotype; however, due to the complexity of the toosendanin structure, and the unlikely possibility that a total synthesis will be developed, an alternative Function-Oriented Synthesis (FOS) [71] strategy has been applied to determine the structural features important for the anti-BoNT activity of this compound. The principle of FOS is that the function of a biologically active lead structure can be emulated, tuned, or possibly improved by replacement with simpler scaffolds designed to encompass the key activity-determining structural features of the natural product. To this end, a CD-ring fragment with an epoxide moiety on the five-member-ring [69] and two epimers of an AB-ring fragment [70] (Figure 4) have been synthesized and tested. 

In a rat spinal cord cellular assay (RSC), addition of two epimers of the AB-ring fragment at 1 mM concentrations did not prevent BoNT/A induced SNAP-25 cleavage in primary neuronal cells, while 200 µM toosendanin (**1**) resulted in complete inhibition of BoNT/A activity. In a mouse lethality assay (MLA), intravenous administration of the synthesized CD-ring fragment compound did not protect or prolong the mean time to death, while at the same concentration toosendanin extended time to death 7.1 h. No *in vitro* assay data was reported for the CD-ring fragment.

The endosome acidification process is required for BoNT-induced muscle paralysis. This is evidenced by the fact that ammonium chloride and methylamine hydrochloride exhibit concentration- and time-dependent antagonism of the onset of neuromuscular blockade by BoNT/A, /B, /C, and tetanus toxin [72,73]. However, these amines act solely by antagonizing the internalization of the toxins by inhibiting endosome acidification, since they neither inactivate the toxins, nor irreversibly change tissue function at concentrations that antagonize the onset of BoNT-induced paralysis. 

**Figure 5:**
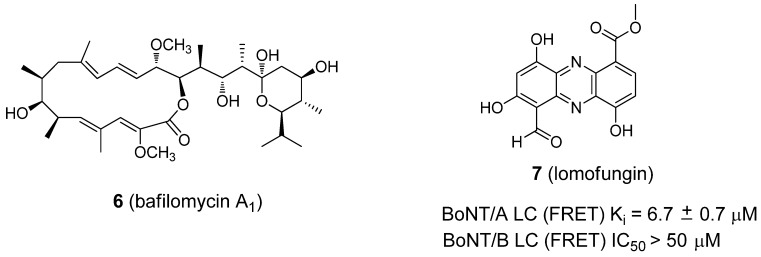
Structures of BoNT inhibitors **6 **(bafilomycin A_1_) and **7** (lomofungin).

Following the same logic, cellular ATPase is required for the acidification of endocytotic vesicles. Thus, the inhibition of a vesicle H^+^-ATPase could result in the antagonism of a broad-spectrum of BoNTs. To this end, bafilomycin A (Figure 5, compound **6**), an ATPase inhibitor, has been shown to be a universal antagonist of BoNTs A-G, as well as tetanus toxin [74]. 

A natural product, lomofungin (**7**, Figure 5) [75], was identified as an inhibitor of the BoNT/A LC (K_i_ value of 6.7 ± 0.7 µM) from the high-throughput screening (HTS) of a drug library. The screening was conducted by The Scripps Research Institute. Lomofungin displayed classical noncompetitive inhibition kinetics and was not mutually exclusive when examined in tandem with an active site inhibitor (2,4-dichlorocinnamic hydroxamate) [76] and a noncompetitive inhibitor, D-chicoric acid. These data suggest that lomofungin binds to a different ligand binding site on the BoNT/A LC. In the same report, lomofungin was reported to display weak BoNT/B LC inhibition (IC_50_ ≥ 50 µM) in a FRET-based assay. 

## 5. Small Molecule BoNT/A LC Inhibitors

**Figure 6:**
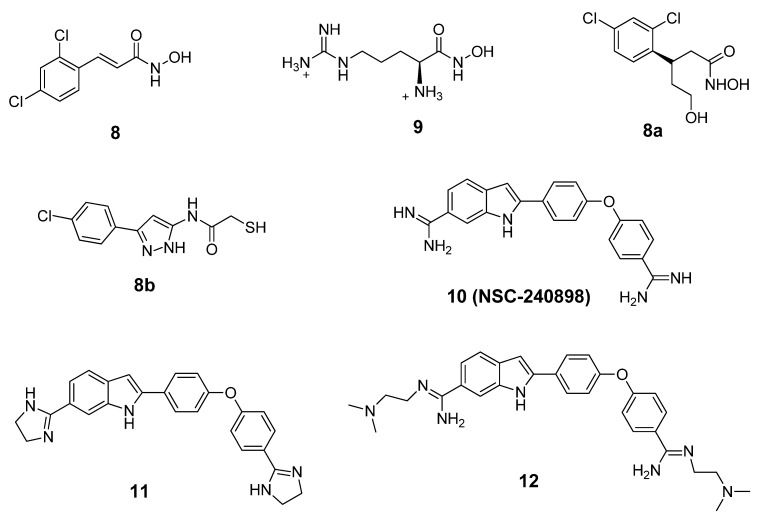
Structures of BoNT/A LC inhibitors.

**Figure 7:**
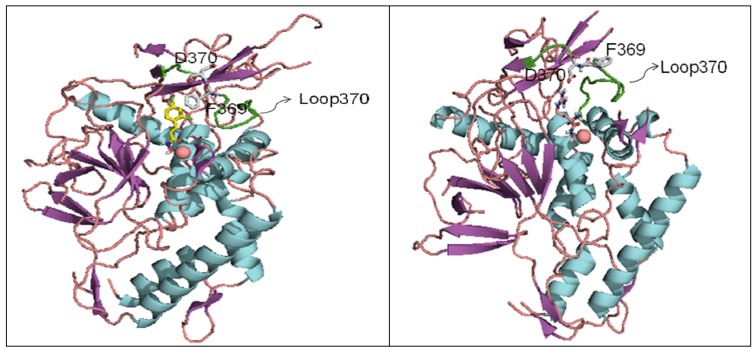
(**a**) Co-crystal structure of BONT/A LC:**8** (PDB code: 2IMA). (**b**) Co-crystal structure of BONT/A LC:**9** (PDB code: 2IMB).

BoNT LCs induce neuronal paralysis via the specific proteolysis of SNARE proteins. Therefore, inhibiting BoNT LC activity has been the major focus of research efforts to discover and develop selective therapeutic agents. Several ligand binding sites have been identified within the BoNT/A LC substrate binding domain, including the active site, a- and b-exosites, and the Cys165 site (¡-exosite). Active site inhibitors usually exhibit competitive kinetics *vs* the SNAP-25 substrate. Co-crystal structures of 2,4-dichlorocinamic hydroxamate (Figure 6, compound **8**) and L-arginine hydroxamate (Figure 6, compound **9**) in complex with the BoNT/A LC have shown that the hydroxamates bind in the enzyme’s active site, with the cinnamyl side chain oriented toward the 370 loop, and the catalytic water molecule (which ligates the zinc ion), displaced by the hydroxamate moiety (Figure 7). 

The hydroxyl oxygen of the hydroxamate moiety coordinates the catalytic zinc ion. However, a dramatic conformational change is observed for the 370 loop in comparing the two complexes. In the complex LC:**9**, the side chain of Phe369 is withdrawn from the catalytic cleft and the side chain of Asp370 is exposed, thereby allowing it to form a salt bridge with the guanidinium functionality of compound **9**. Rational design based on this co-crystal data resulted in the identification of chiral compound **8a,** which possesses an R-configuration at the b-carbon. Compound **8a** displayed a K_i_ value of 0.16 mM ± 0.002 µM [77]. Moe *et al*. [78] have reported a series of mercaptoacetamides, for example compound **8b**, which provide low μM anti-BoNT/A LC activity. The SAR of the mercaptoacetamides is very similar to that of the cinnamic hydroxamates, suggesting that these inhibitors also bind in the BoNT/A LC catalytic cleft.

NSC-240898 (Figure 6, compound **10**), a bisamidine compound, has been identified as a BoNT/A LC inhibitor from screening a diversity set of small molecules from the National Cancer Institute’s Open Repository (using a high throughput FRET-based enzyme assay) [79,80]. Chemical optimization studies of this lead structure have been conducted at both the University of Pittsburgh [81] and Microbiotix, Inc. [54]. 

**Figure 8:**
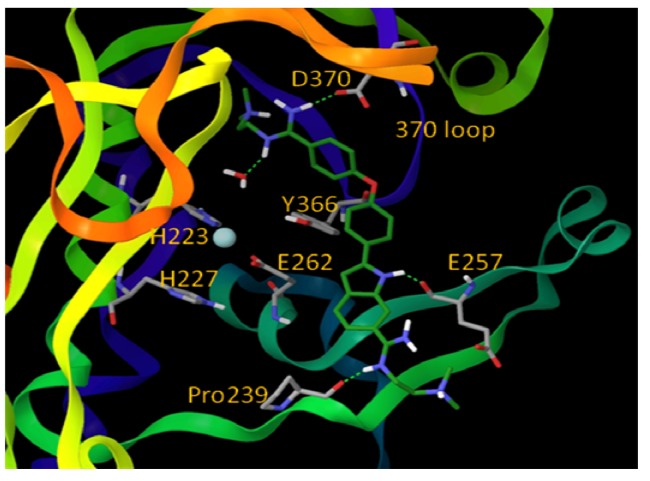
Proposed binding mode for inhibitor **12** (shown in green stick model). Oxygen atoms are red, nitrogen atoms are blue, hydrogen atoms are white, and Zn is cyan. The BoNT/A LC is rendered ribbon. (Reproduced from Li, B. *et al*. *J. Med. Chem*.; published by American Chemical Society [54].)

One of the most potent analogs of this chemotype is compound **12** (Figure 6), which possesses an IC_50_ value of 2.5 µM in a FRET-based enzyme assay. Hence, **12** is 4.4-fold more potent than the lead structure (IC_50_ = 11 µM), 3-fold more potent than cinnamic acid hydroxamate **8** (IC_50_ = 8.9 µM), and 11.2-fold more selective for the BoNT/A LC than anthrax lethal factor (also a metalloprotease). Compound **11**, another analog of NSC-240898, possesses IC_50_ values of 12.5 µM and 9.4 µM in a FRET-based and an HPLC-based assay, respectively, and has shown protection against BoNT/A-induced cleavage of SNAP-25 in both rat and chicken neuronal cell-based assays [82]. Bisamidine **10** and its analogs are competitive BoNT/A LC inhibitors, and molecular modeling studies suggest that they bind in the active site of the BoNT/A LC and do not directly interact with the catalytic zinc ion (Figure 8).

**Figure 9:**
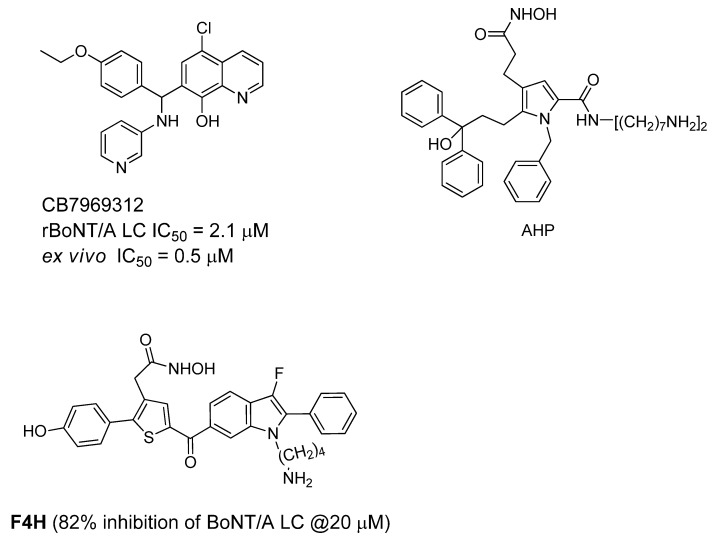
Active site BoNT/A LC inhibitors.

Recently, an *in silico* screening campaign, coupled with biochemical assays, has been used to identify BoNT/A LC inhibitors. From this study, quinolinol derivative CB7969312 (Figure 9), has been reported as a potent inhibitor that protects against neuromuscular block in an *ex vivo* mouse phrenic nerve hemidiaphram assay (EC_50_ = 0.5 µM). 

This compound also significantly neutralizes the BoNT/A holotoxin in N2a cells [83]. Biochemical analysis of the inhibition and binding of this quinolinol compound with the BoNT/A LC suggest that it exhibits atypical noncompetitive kinetics [84]. A molecular docking study suggests that the quinolinol compound binds within a large hydrophobic pocket in the BoNT/A LC active site, and that the hydroxyquinoline moiety purportedly binds the catalytic zinc. Pang *et al*. [85] reported a series of competitive, active site inhibitors based on pyrrole and thiophene structures using synthesis-based computer-aided molecular design. The most potent compound (AHP) displayed BoNT/A inhibition with a K_i_ value of 0.76 ± 0.17 µM and an IC_50_ value of <1 µM, and a thiophene-based compound (F4H) demonstrated 100% and 70% protection of mice against BoNT/A at 5ED_50_ within periods of two and four half-lives, respectively, of the inhibitors [86]. In the study, a single dose of inhibitor was administered IP (concentration = 2 mg/kg) pre-BoNT challenge.

**Figure 10:**
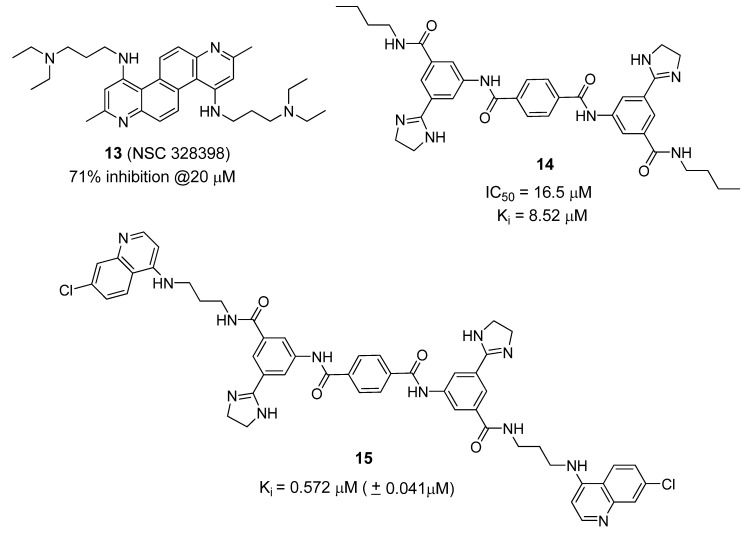
Small molecule BoNT/A LC inhibitors identified using phamacophore-based design.

Several non-Zn-chelating small molecule (non-peptidic) BoNT/A LC inhibitors (SMNPIs) have been described based on pharmacophore-based design [80,87,88,89]. SMNPIs are continually integrated into the pharmacophore to both develop three-dimensional (3D) search queries to discover novel SMNPI chemotypes and guide the rational design of more potent SMNPI derivatives. Employing this iterative approach, new chemotypes including diazachrysene (Figure 10, compound **13**) and phenylterephthalamide (Figure 10, compound **14**), have been identified. The pharmacophore model has guided design and synthesis of compound **15** (Figure 10), which possesses a K*_i_* value of 0.572 µM (± 0.041 µM) *vs*. the BoNT/A LC.

**Figure 11:**
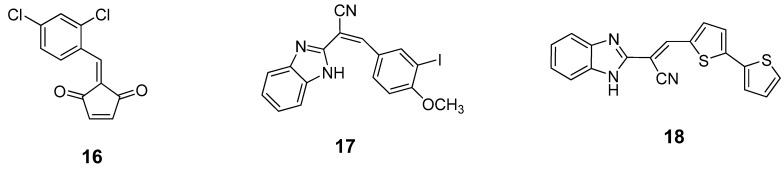
Covalent inhibitors of BoNT/A LC.

A series of benzylidene cyclopentenedione-based inhibitors has been reported to inhibit the BoNT/A LC metalloprotease by putatively forming a covalent bond with the enzyme [90]. Among these inhibitors, compound **16** (Figure 11) displayed a *k_inact_*/K_I_ value of 520 M^-1^s^-1^ and SNAP-25 cleavage was significantly decreased at concentrations of 600 µM in a primary rat spinal cord neuron assay. Unfortunately, compound **16** is highly bound to serum and is reactive with glutathione. Such a poor pharmacokinetic profile prevents further development of this type of inhibitor. Researchers at Microbiotix have identified benzimidazole compound **17** (Figure 11) from high throughput, FRET-based screening of compound libraries against the BoNT/A LC. Compound **17** inhibited the BoNT/A LC with an IC_50_ value of 7.2 µM in the FRET-based assay (and 10 µM in an HPLC-based assay); however, no cell-based activity was observed.

**Figure 12:**
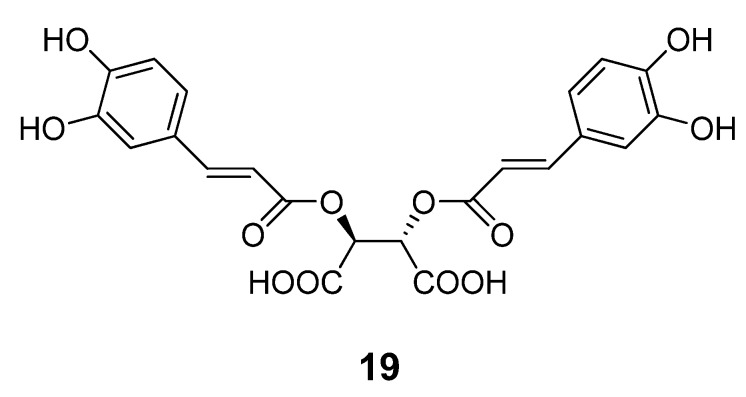
Chemical structure of D-chicoric acid (**19**).

Chemical optimization of the benzimidazole acrylonitrile series of BoNT/A LC inhibitors yielded compound **18**, which possesses an IC_50_ value of 26 µM. Moreover, compound **18** displays 58% protection of SNAP-25 cleavage at an inhibitor concentration of 30 µM [57]. Silhar *et al.* reported that a natural product isolated from Echinacea, D-chicoric acid (**19**) (Figure 12), inhibits BoNT/A LC activity by binding to an exosite, and displays noncompetitive partial inhibition of the LC with a submicromolar inhibition constant [76]. In a combination study, D-chicoric acid was synergistic with competitive BoNT/A LC inhibitor 2,4-dichlorocinnamic hydroxamic acid (**8**).

## 6. Small Molecule BoNT/B LC Inhibitors

**Figure 13:**
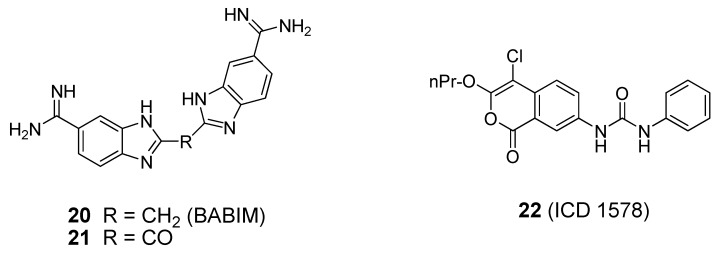
Small molecule BoNT/B LC inhibitors.

Known zinc chelators, such as bis(5-amidino-2-benzimidazolyl)methane (BABIM, Figure 13, compound **20**), have been shown to be weak BoNT/B LC inhibitors (IC_50_ = 5-10 µM) [91]. X-ray co-crystal structures of BABIM in complex with the BoNT/B LC have been reported [39,91]. As shown in the latter published BABIM:BoNT/B structure, two inhibitor molecules are bound to the holotoxin. One molecule of BABIM enters through a cleft formed between the translocation domain and the catalytic domain. Another molecule of BABIM sits in the cleft formed between the translocation domain and the binding domain, suggesting that there are two pathways for the inhibitor to enter the toxin. The two inhibitor molecules do not bind to the enzyme’s catalytic zinc, as was observed in a previously reported BoNT/B LC:BABIM structure [39]. The co-crystal structures suggest that, in the presence of inhibitor, the environment of the active site rearranges, and the catalytic zinc is gradually removed from the active site and transported to a different site of the protein. ICD 1578 (**22**, Figure 12), a human leukocyte elastase inhibitor, was reported to inhibit the BoNT/B LC with an IC_50_ value of 27.6 µM in a FRET-based assay using a 50-mer synaptobrevin peptide as substrate [92]. However, no enzyme selectivity data has been presented for compound **22**. To date, BoNT/B LC specific small molecule inhibitors suitable for clinical use have not been reported.

## 7. Summary

As described above, large molecule biologics such as monoclonal antibodies and receptor decoys may be effective in mouse models or cellular systems if they are administered simultaneously with BoNT; however, they have not been tested for, and are unlikely to be useful for, post-exposure therapy (*i.e.*, after BoNT penetration into the neuronal cytosol). Peptide-based inhibitors suffer from a similar limitation because their large molecular size and metabolic instability limit their ability to reach the BoNT endopeptidase within neurons. Therefore, small molecule, non-peptidic inhibitors offer the best opportunity for the development of post-exposure therapeutics. However, none of the reported agents demonstrate adequate therapeutic utility and none have shown protection in mice due to limited efficacy, poor cell membrane permeability, cytotoxicity, or poor pharmacokinetic properties, although some of them may have prolonged the mean time to death. Thus, more drug-like small molecule botulinum inhibitors, which are potent, effective, safe, and possess suitable absorption, distribution, metabolism, excretion, and toxicity (ADMET) profiles are urgently needed. The key would be to further refine the design strategy to develop analogs of the lead molecules with improved solubility or ADMET properties. Viable biological assays to target different phases of BoNT intoxication are also needed to identify inhibitors with novel mechanisms of action. The development of high-throughput screening enzymatic and cell-based assays, as well as structure-based drug design approaches, are valuable for the identification of inhibitor ‘leads’ for further optimization. Finally, the validation of *in vivo* models of BoNT intoxication will be important for the determination of compounds that will be of clinical use.
